# A Self-Report Risk Index to Predict Occurrence of Dementia in Three Independent Cohorts of Older Adults: The ANU-ADRI

**DOI:** 10.1371/journal.pone.0086141

**Published:** 2014-01-23

**Authors:** Kaarin J. Anstey, Nicolas Cherbuin, Pushpani M. Herath, Chengxuan Qiu, Lewis H. Kuller, Oscar L. Lopez, Robert S. Wilson, Laura Fratiglioni

**Affiliations:** 1 Centre for Research on Ageing, Health and Wellbeing, The Australian National University, Canberra, Australia; 2 Aging Research Center, Karolinska Institutet-Stockholm University, Stockholm, Sweden; 3 Department of Epidemiology, University of Pittsburgh, Pittsburgh, Pennsylvania, United States of America; 4 Department of Neurology, University of Pittsburgh, Pittsburgh, Pennsylvania, United States of America; 5 Rush Alzheimer's Disease Center, Rush University Medical Center, Chicago, Illinois, United States of America; 6 Stockholm Gerontology Research Center, Stockholm, Sweden; University of Nebraska Medical Center, United States of America

## Abstract

**Background and Aims:**

The Australian National University AD Risk Index (ANU-ADRI, http://anuadri.anu.edu.au) is a self-report risk index developed using an evidence-based medicine approach to measure risk of Alzheimer's disease (AD). We aimed to evaluate the extent to which the ANU-ADRI can predict the risk of AD in older adults and to compare the ANU-ADRI to the dementia risk index developed from the Cardiovascular Risk Factors, Aging and Dementia (CAIDE) study for middle-aged cohorts.

**Methods:**

This study included three validation cohorts, i.e., the Rush Memory and Aging Study (MAP) (n = 903, age ≥53 years), the Kungsholmen Project (KP) (n = 905, age ≥75 years), and the Cardiovascular Health Cognition Study (CVHS) (n = 2496, age ≥65 years) that were each followed for dementia. Baseline data were collected on exposure to the 15 risk factors included in the ANU-ADRI of which MAP had 10, KP had 8 and CVHS had 9. Risk scores and C-statistics were computed for individual participants for the ANU-ADRI and the CAIDE index.

**Results:**

For the ANU-ADRI using available data, the MAP study c-statistic was 0·637 (95% CI 0·596–0·678), for the KP study it was 0·740 (0·712–0·768) and for the CVHS it was 0·733 (0·691–0·776) for predicting AD. When a common set of risk and protective factors were used c-statistics were 0.689 (95% CI 0.650–0.727), 0.666 (0.628–0.704) and 0.734 (0.707–0.761) for MAP, KP and CVHS respectively. Results for CAIDE ranged from c-statistics of 0.488 (0.427–0.554) to 0.595 (0.565–0.625).

**Conclusion:**

A composite risk score derived from the ANU-ADRI weights including 8–10 risk or protective factors is a valid, self-report tool to identify those at risk of AD and dementia. The accuracy can be further improved in studies including more risk factors and younger cohorts with long-term follow-up.

## Introduction

Alzheimer's dementia (AD) affects approximately 35.6 million people worldwide, and this will increase with population ageing [1]. There is increasing focus on delaying the onset of AD through intervening to modify risk factors [2], [3], and it has been estimated that a 10–25% reduction in seven key risk factors could prevent 1·1–3·0 million AD cases internationally [2]. Hence, it is useful to have scientifically based tools that measure individuals' risk factor profiles associated with developing late-life AD.

Development of risk assessment tools poses several challenges in the field of dementia and more generally. Most often, tools are developed by analysing a single cohort and identifying a set of predictors from that individual study using logistic regression and receiver operating characteristics to identify optimal cut-offs. This approach has been used in many areas of health and medicine, such as developing falls risk indices, the Framingham risk score, a risk index for identifying unsafe drivers, and risk indices for dementia. The main limitation of this approach is that the risk index is optimised for the study from which it was derived. Without external validation, it is not possible to know how generalizeable such risk indices are. An alternative approach is to develop a risk index based on synthesis of information about risk factors derived from multiple cohort studies. To date we know of few examples of this in the literature. This latter approach requires that sufficient data have been published relating to individual risk factors.

Using an Evidence-Based Medicine approach involving data synthesis we developed the Australian National University AD Risk Index (ANU-ADRI) [4], to identify the degree to which individuals are ‘at-risk’ of AD based on the risk factors identified in epidemiological studies that could be measured using self-report. This assessment tool differs from previous tools developed to predict dementia because it was not developed by identifying risk factors from a single cohort study and does not include any variables that require clinical assessment or laboratory tests.

In studies reporting the general outcome of ‘dementia’ as well as specific subtypes of dementia such as AD, or Vascular Dementia, results for ‘dementia’ are often similar to that of AD because AD accounts for up to 75% of all dementia cases. Hence, although we developed the ANU-ADRI from literature reporting effects for AD specifically, we evaluate the tool against both diagnoses of AD and the more general diagnostic outcome of dementia. If it predicts both outcomes, it will have a wider utility.

The aim of the present study is to evaluate the ANU-ADRI by estimating the associations between ANU-ADRI and incident AD and dementia in three cohorts from two countries (USA and Sweden). Second, we aimed to evaluate whether the ANU-ADRI had improved capacity to predict AD and dementia compared with a previously published dementia risk score developed on a single cohort [5] based on midlife assessments of risk factors. This would provide information on the utility of that measure used in older cohorts, and the capacity of the ANU-ADRI to contribute to the range of available low-cost dementia risk assessment tools.

## Methods

### The ANU-ADRI

The development of the ANU-ADRI has been previously described and is summarised here briefly [4]. An Evidence-based Medicine Approach was used to identify risk and protective factors for AD that could be measured by self-report. We systematically searched the literature and identified 11 risk factors (age, sex, low education, diabetes, traumatic brain injury, depressive symptoms, smoking, low social networks) and four protective factors (cognitively stimulating activities, alcohol consumption, physical activity, fish intake) for AD for which pooled estimates of risk ratios had been published or could be estimated from high quality articles meeting criteria used in previous publications [13]–[16]. The definitions of variables in the ANU-ADRI algorithm were derived from the exposure variables used in meta-analyses from which risk ratios were derived, except where this was not possible. The points for each factor were derived from beta-weights from converted published odds ratios using the method described previously [5]. To create an integer level scoring system, scores were multiplied by a constant. Individual ANU-ADRI scores were created by an algorithm that sums the points attributed to individual risk and protective factors using an additive method [5], [17].

For each of the three cohort studies, all predictive variables included in the ANU-ADRI were selected from their baseline or from the first occasion at which the risk factor was measured. Table 1 shows the measures used for each risk and protective factor in each cohort. They were coded as categorical variables and assigned a score according to the point system described above.

**Table 1:** Risk and protective factors included from each cohort.

Risk/protective factor	MAP	KP	CVHS	ANU-ADRI
Age and gender	Self report	Self report	Self report	Self report
Education	Number of years	Number of years	Number of years	Scale was created using number of years
Diabetes	History of diabetes and medication	History of diabetes, medication and blood test	History of diabetes, medication and blood test	History of diabetes and medication
Traumatic Brain Injury	History for TBI with loss of consciousness	History for TBI with loss of consciousness	NA	History for TBI with loss of consciousness
Cognitive activity	A structured interview focused on cognitive activities in late life	NA	NA	The questionnaire developed for MAP
Social network and engagement	5 domains (marital status, size of social network, quality of social network, level of social activities and living arrangements)	5 domains (marital status, size of social network, quality of social network, level of social activities and living arrangements)	NA	5 domains (marital status, size of social network, quality of social network, level of social activities and living arrangements)
Smoking	Questions for smoking statues (current smoking, ever smoking and never smoking)	Only asked for current smoking and never smoking)	Questions for smoking status (current smoking, ever smoking and never smoking)	Questions for smoking status (current smoking, ever smoking and never smoking)
Alcohol	Categories were calculated according to NHMRC guidelines using number of drinks per week.	The scale in the variable included non-alcoholics and light to moderate alcoholics.	Categories were calculated according to NHMRC guidelines using number of drinks per week.	Categories were calculated according to NHMRC guidelines using number of drinks per week.
Physical activity	Minnesota Leisure Time Physical Activity Questionnaire: categories were calculated using MET	NA	Minnesota Leisure Time Physical Activity Questionnaire: categories were calculated using MET	International Physical Activity Questionnaire (IPAQ) categories were calculated using MET
Fish intake	NA	NA	Modified National Cancer Institute FFQ	National Cancer Institute FFQ
Depression symptoms	NA	NA	CES-D (10 item) >11 was used as cutoff	CES-D (20 item) >16 was used as cutoff

NHMRC- Australian National Health and Medical Research Council, MET- Metabolic Equivalent, FFQ- Food Frequency Questionnaire, CES-D- Centre for Epidemiology Scale for Depression. NA = Not available or data not compatible with ANU-ADRI scoring.

We excluded BMI and cholesterol measures because these factors, when measured in late life (60 years and older), have not been conclusively associated with increased risk of AD [13], [14]. Participants diagnosed with dementia at baseline were excluded from analysis as were participants who had missing data for diagnosis of AD or dementia at last follow-up.

### Validation samples

We reviewed the literature to identify high quality longitudinal studies including a large proportion of the risk factors included in the ANU-ADRI, and longitudinal follow-up for AD and dementia. No study was identified that included all the risk and protective factors included in the ANU-ADRI, and that had dementia diagnoses. Three studies were identified with nine or more risk or protective factors and were available for analysis by the study owners.

The Rush Memory and Aging Project (MAP) comprised 1164 participants aged 53 years and older who were initially assessed in 1997 and followed for an average of 3·5 years. The study design and details of dementia diagnosis have been previously published [6]. The diagnosis of dementia and AD were established by experienced physicians using the National Institute of Neurological and Communicative Diseases and Stroke–Alzheimer's Disease and Related Disorders Association (NINCDS-ADRA) criteria for diagnosis of AD. For the MAP project, Age, Gender, Education, Diabetes, Traumatic Brain Injury, Cognitive Stimulating Activities, Social Engagement, Smoking, Alcohol, Physical Activity were included in the computation of the ANU-ADRI. The possible range of the ANU-ADRI scores was −13 to 64. There were 903 participants with complete data used in the analysis of MAP at baseline.

The Kungsholmen Project (KP) comprised 1301 participants initially aged 75 years and older who were assessed in 1987–1989 and followed in 1991–1993 and 1994–1996 for an average of 6 years. Details of the design and diagnosis of dementia have also been published previously [7], [8]. The baseline dementia-free cohort was determined using a two-phase procedure, that is, a screening phase for all participants (n = 1810) with a structured interview and the administration of Mini-Mental State Examination (MMSE), followed by a clinical phase for all those with MMSE score <24 and an age- and sex-matched random sample of those with MMSE score ≥24. AD and dementia were diagnosed according to DSM-III-R criteria by physicians using a validated three-step diagnostic procedure [9]. The algorithm for the KP included Age, Gender, Education, Diabetes, Traumatic Brain Injury, Social Engagement, Smoking, Alcohol Consumption. The physical and cognitive activity measures were not comparable to those included in the ANU-ADRI and hence could not be included. The potential range of ANU-ADRI scores was −3 to 61. A total of 905 participants had complete data on risk factors and were included in the present study.

The Cardiovascular Health Cognition Study (CVHS) was an ancillary study of a larger Cardiovascular Health Study which was initiated in 1989–1990 with 5201 primarily Caucasian adults aged 65 years and older. In the fifth year of the study 687 African American adults were added [10]–[12]. The CVHS was initiated in 1998–1999 with 3606 subjects who had a cerebral MRI and Modified MMSE in 1991–1994. For the present study participants who had dementia at baseline were excluded so the potential sample for analysis included 3375 participants followed for an average of 6 years. Dementia was diagnosed based on a progressive or static cognitive deficit of sufficient severity to affect the participant's activities of daily living in at least two cognitive domains, which did not necessarily include memory. As previously described, type of dementia was classified as probable or possible AD (NINCDS-ADRDA), probable or possible vascular dementia (State of California Alzheimer's Disease Diagnostic and Treatment Centers criteria), mixed dementia, or other dementia. MRI findings were used only to aid in classification of dementia but not in the initial dementia diagnosis. All dementia cases were assessed and confirmed by expert neurologists and psychiatrists. The baseline assessment included nine of the fifteen factors in the ANU-ADRI (Age, Gender, Education, Diabetes, Depressive symptoms, Smoking, Alcohol, Physical Activity, Fish Intake) yielding a potential range of the ANU-ADRI scores of −11 to 56. There were 2496 participants with complete data on the ANU-ADRI.

All three studies received approval from their Institutional Review Boards. The ANU-ADRI study protocol was approved by the Human Research Ethics Committee at the Australian National University, Canberra (protocol number: 2011/064). Datasets are available from the authors affiliated with each study and the syntax for this study is available from KJA and MH.

### The Cardiovascular Risk Factors, Aging and Dementia (CAIDE) Index

The CAIDE index was developed through analysis of the CAIDE cohort study (n = 1409) which is one of the few studies to measure risk factors in midlife and follow participants until late life and obtain dementia diagnoses [5]. The CAIDE risk score included a subset of items included in the ANU-ADRI although they were defined according to the data available in the CAIDE baseline assessment. The CAIDE risk index included age (>47 years), sex (female), low education (<10 years), hypercholesterolaemia (>6.5 mmol/L), high systolic blood pressure (>140 mm Hg), physical activity (active versus non-active) and obesity (BMI≥30 kg/m^2^). A CAIDE equivalent score was calculated for MAP, KP and CVHS. Weights were applied to each risk factor as previously reported [5]. A CAIDE score was also calculated by excluding BMI and BMI and cholesterol because of the older age of the validation cohorts.

### Statistical analyses

The accuracy of the statistical models for identifying participants at risk of AD using the ANU-ADRI was quantified by calculating the area under the (AUC), c-statistics, and 95% confidence interval for each study. The c-statistic integrates the measures of sensitivity and specificity of the variables included in the model with a value of 1·00 being associated with perfect predictive value and a value of 0·50 or less being associated with chance. The predictive accuracy of the proposed statistical model was further evaluated for each gender and for the outcome of any dementia.

The ANU-ADRI score was calculated by adding points allocated to individual risk/protective factors. The methodology for the scoring system has been previously published in detail [4]. Protective factors had negative weights indicating their association with reduced risk of AD or dementia. For each study, the distribution of ANU-ADRI scores was divided into quartiles within each study. Cumulative hazards ratios for conversion to AD were estimated for each quartile using cox regression, and incidence rates for each quartile of the ANU-ADRI scores were estimated based on the observed data.

To assess whether results were biased due to missing data within each dataset, analyses were also run using multiple imputation (MI) with 10 datasets using the MI procedure in SPSS which estimates the pooled effects based on analyses of the imputed datasets [18]. Results from analyses using the imputed datasets did not differ significantly from those of the original datasets (data not shown). Results reported were based on complete cases. To provide comparison of the accuracy of the ANU-ADRI with an index derived from a single cohort study, c-statistics were also calculated for the MAP, KP and CVHS cohorts for the risk index developed on the CAIDE studies [5].

## Results

### Description of the three validation samples

Table 2 shows the characteristics of the evaluation cohorts and the frequency of the individual risk factors within each study cohort using the categories for each risk factor included in the ANU-ADRI. MAP was the only study to include participants classified in the midlife range (aged between 40 and 60) but this only represented 2% of the total sample. The KP sample was older than that of the other two studies at baseline. The three samples differed in the prevalence of several risk factors and in the inclusion of risk factors measured at baseline. Over 90% of the MAP participants reported having more than 11 years of education. The KP cohort included a large proportion of participants with fewer than eight years of education, The proportions of incident dementia cases occurring during the follow-up periods were highest in the MAP and KP cohorts; the proportions of incident AD were 17·5% for MAP, 20·0% for KP and 11·1% for CVHS. The points attributed to each of the risk and protective factors are also reported in Table 2.

**Table 2:** Descriptive statistics for the three evaluation cohorts, their measured risk and protective factors, and the points allocated to each factor on the ANU-ADRI.

		MAP (n = 1164)	KP (n = 1301)	CVHS (n = 3375)	Points
Location of study population		United States	Sweden	United States	
Age at baseline (years), mean (SD)		79·8 (7·4)	81·5 (5·0)	72·3 (4·9)	
Range of age (years)		54–100	74–100	62–95	
Gender: Male, n (%)		300 (24·8)	325 (25·0)	1381 (40.9)	
Age for males (years), n (%)	<65	6 (0.5)	0	30 (0.9)	0
	65–69	12 (1)	0	356 (10.5)	1
	70–74	39 (3.4)	6 (0.5)	607 (18.0)	12
	75–79	69 (5.9)	141 (10.8)	263 (7.8)	18
	80–84	104 (8.9)	106 (8.1)	86 (2.5)	26
	85–89	53 (4.6)	50 (3.8)	24 (0.7)	33
	≥90	17 (1.5)	22 (1.7)	3 (0.1)	38
Age for females (years), n (%)	<65	40 (3.4)	0	62 (1.8)	0
	65–69	73 (6·3)	0	626 (18·5)	5
	70–74	98 (8·4)	15 (1·2)	787 (23·3)	14
	75–79	199 (17·1)	376 (28·9)	355 (10·5)	21
	80–84	24·7 (21·2)	306 (23·5)	123 (3·6)	29
	85–89	149 (12·8)	194 (14·9)	18 (0·5)	35
	≥90	57 (4·9)	85 (6·5)	1 (0)	41
Educational level (years), n (%)	<8	42 (3·5)	654 (50·3)	367 (11·0)	0
	8–11	60 (5·0)	253 (19·4)	439 (13·1)	3
	>11	1061 (87·8)	389 (29·9)	2537 (75·9)	6
Diabetes, n (%)	No	1016 (94·4)	1187 (91·2)	2805 (83·1)	0
	Yes	147 (12·2)	114 (8·5)	537 (16·0)	3
Traumatic Brain Injury, n (%)	No	1098 (94·4)	877 (67·4)	NA	0
	Yes	65 (5·4)	86 (6·6)	NA	4
Depressive symptoms, n (%)	No	NA	NA	2811 (83·3)	0
	Yes	NA	NA	180 (5·3)	2
Cognitively stimulating activities, n (%)	Low	408 (33·8)	NA	NA	0
	Moderate	600 (49·7)	NA	NA	−6
	High	155 (12·8)	NA	NA	−7
Social network, n (%)	High	97 (8·0)	13 (1·0)	NA	0
	Medium-high	328 (27·2)	226 (17·4)	NA	1
	Medium-low	422 (34·9)	880 (67·6)	NA	4
	Low	125 (10·3)	84 (6·5)	NA	6
Smoking, n (%)	Never smoked	685 (56·7)	867 (66·7)	1558 (46.5)	0
	Former smoker	432 (35·8)	NA	1426 (42.6)	1
	Current smoker	44 (3·6)	104 (8·0)	364 (10.9)	4
Alcohol consumption, n (%)	Abstainers	193 (16·0)	391 (30·1)	1585 (47·3)	0
	Light-to-moderate	887 (74·3)	577 (44·4)	1634 (48·8)	−3
	Heavy	80 (6·6)	0	129 (3·9)	
Physical activity, n (%)	Low	465 (38·5)	NA	993 (29·7)	0
	Medium	495 (41·0)	NA	1677 (50·2)	−2
	High	203 (16·8)	NA	671 (20·1)	−3
Fish intake (serves/week), n (%)	0–0·25	NA	NA	388 (13·2)	0
	0·26–2·0	NA	NA	1168 (39·8)	−3
	2·1–4·0	NA	NA	1263 (43·0)	−4
	≥4·1	NA	NA	119 (4·1)	−5

Note. NA = Data not available either because it was not collected or collected but not in a format compatible with the ANU-ADRI. The points are the weights given to each level of each variable in the ANU-ADRI risk score.

### Results of predictive analyses in each cohort

Table 3 reports the results of the analyses evaluating the accuracy of the ANU-ADRI for predicting AD and dementia in each cohort. C-statistics for the ANU-ADRI predicting AD were 0·733 (95% CI 0·691–0·776) for the MAP study, 0·637 (95% CI 0·596–0·678) for the KP study, and 0·740 (95% CI 0·712–0·768) for the CVHS study. The number of risk/protective factors included for each study was 10, 8 and 9 for MAP, KP and CVHS respectively. The ANU-ADRI risk score quartiles for individual studies with their median values are also presented in Table 4. The higher scores for each quartile of the KP study reflect the older age of this cohort. When only common variables among studies were included in analyses (age, sex, education, diabetes, smoking and alcohol) the c-statistics were 0.734 (95% CI 0.707–0.761) for the CVHS, 0.689 (0.650–0.727) for the MAP and 0.666 (0.628–0.704) for the KP studies.

**Table 3:** Characteristics and accuracy of ANU-ADRI for predicting AD and dementia in the three cohort studies.

	MAP (n = 903)	KP (n = 905)	CVHS (n = 2496)
Follow-up (years)	Mean 3.5 (SD 3.0)	Mean 6.0 (SD 5.7)	Median 6.0
Risk and protective factors	(10) Age, Gender, Education, Diabetes, TBI, Cognitive Stimulating Activities, Social Engagement, Smoking, Alcohol, Physical Activity	(8) Age, Gender, Education, Diabetes, TBI, Social Engagement, Smoking, Alcohol	(9) Age, Gender, Education, Diabetes, Depression, Smoking, Alcohol, Physical Activity, Fish Intake
**ANU-ADRI score**			
Mean (SD)	20·3 (11·78)	32.38 (7·67)	8.86 (8.77)
Quartiles, median (range)			
Quartile 1	6·5 (−11·0–13·0)	22·0 (13·0–27·0)	−2.0 (−10.0–3·0)
Quartile 2	18·0 (13·1–21·0)	30·0 (27·1–31·0)	6·0 (3.1–8·0)
Quartile 3	25·0 (21·1–29·0)	36·0 (31·1–37·0)	11·0 (8.1–14·5)
Quartile 4	34·0 (29·1–46·0)	42·0 (37·1–57·0)	19·0 (14.51–41·0)
**Alzheimer's Disease**			
AUC for men	0·715 (0·630–0·799)	0·672 (0·586–0·759)	0·732 (0·686–0·778)
AUC for female	0·744 (0·695–0·793)	0·620 (0·574–0·667)	0·742 (0·707–0·778)
AUC overall	0·733 (0·691–0·776)	0·637 (0·596–0·678)	0·740 (0·712–0·768)
AUC for common variables*	0.689 (0.650–0.727)	0.666 (0.628–0.704)	0.734 (0.707–0.761)
**Any dementia**			
AUC for men	0·714 (0·630–0·797)	0·728 (0·655–0·801)	0·713 (0·670–0·756)
AUC for female	0·728 (0·679–0·778)	0·626 (0·582–0·670)	0·738(0·704–0·772)
AUC overall	0·721 (0·678–0·764)	0·653 (0·616–0·691)	0·728 (0·701–0·754)
AUC for common variables*	0.681 (0.644–0.719)	0.677 (0.642–0.711)	0.723 (0.698–0.749)

Note. The results for quartiles are the mean score within each quartile, the range of scores within each quartile of the observed ANU-ADRI scores within each cohort.

* Common variables for three studies included; age, sex, education, diabetes mellitus, smoking, and alcohol.

**Table 4:** Incidence of AD (per 1000 person-years) and hazards ratios for AD by quartile of ANU-ADRI score.

ANU-ADRI score (quartile)	MAP (n = 890)		KP (n = 904)		CVHS (n = 740)*	
	Incidence	Hazard ratio (95% CI)	Incidence	Hazard ratio (95% CI)	Incidence	Hazard ratio (95% CI)
Quartile 1	12.79	Reference	13.88	Reference	30.16	Reference
Quartile 2	26.17	1.99 (0.92–4.28)	28.46	2.02 (1.24–3.30)	38.13	1.36 (0.84–2.18)
Quartile 3	63.51	4.92 (2.50–9.70)	53.70	3.61 (2.30–5.66)	56.39	2.13 (1.36–3.23)
Quartile 4	86.73	6.71 (3.45–13.06)	59.42	4.00 (2.51–6.31)	92.25	3.86 (2.55–5.90)
P for linear trend	<0.001		<0.001		<0.001	

* There was a large amount of missing data for the CVHS on length of follow-up.

### Comparison of quartiles on the ANU-ADRI

To enable comparison of predictive validity between studies, the incidence rates of AD (per 1000 person-years) were calculated by quartile (Table 4). The incidence rate was significantly increased with the increase of ANU-ADRI score in each quartile (p for linear trend <0·001) demonstrating a dose-response relationship between the ANU-ADRI and incident AD.

### Comparison of adults aged <70 with those ≥70 on the ANU-ADRI

To evaluate the ANU-ADRI in adults aged younger than 70 years the CVHS sample was divided into two groups according to age (<70 vs. ≥70 years) and the percentage of dementia cases per quartile were compared. Table 5 shows that among younger participants, the rates of dementia within the same quartile of risk were the same except for Quartile 3 where they were significantly lower.

**Table 5:** Dementia cases per quartile score on the ANU-ADRI for young-old and old-old in the CVHS study.

Quartile of ANU-ADRI score	ANU-ADRI score range	Age range 65–69			Age range 70–95			?^2^, p-value
		n	Dementia cases	%	n	Dementia Cases	%	
Quartile 1	−10 to 3	649	114	18	100	22	22	1.817, ns
Quartile 2	3.01 to 8	328	65	20	344	93	27	0.350, ns
Quartile 3	8.01 to 14.5	186	47	25	523	163	31	13.350, p<0.001
Quartile 4	14.51 to 42	44	16	36	517	220	43	0.178, ns

### C-statistics for the CAIDE Dementia Risk Score

To enable comparison of the ANU-ADRI with another published risk score we applied the dementia risk score developed from CAIDE study to the MAP, KP and CVHS cohorts. Table 6 shows the c-statistics for the CAIDE index for each study using available risk factors, and also excluding cholesterol, and excluding cholesterol and BMI. The c-statistics were higher excluding cholesterol and BMI, and ranged from 0.552 to 0.584 for AD, and from 0.549 to 0.595 for dementia.

**Table 6:** Characteristics and accuracy of CAIDE for predicting AD and dementia in the three cohort studies.

	MAP (n = 903)	KP (n = 905)	CVHS (n = 2496)
**Alzheimer's Disease**			
AUC overall	0·491 (0·427–0·554)	0·533 (0·487–0·580)	0·568 (0·536–0·600)
AUC excluding BMI	0·543(0·482–0·605)	0·529 (0·485–0·573)	0·584 (0·552–0·610)
AUC excluding BMI & cholesterol	0·552 (0·509–0·594)	N/A*	0·584 (0·552–0·616)
**Any dementia**			
AUC overall	0·488 (0·426–0·549)	0·538 (0·496–0·579)	0·570 (0·541–0·600)
AUC excluding BMI	0.540 (0.480–0.599)	0·536 (0.497–0.574)	0·589(0·560–0·619)
AUC excluding BMI & cholesterol	0·549 (0·507–0·591)	N/A*	0·595 (0·565–0·625)

* cholesterol data were unavailable for the KP study.

## Discussion

We report the first evaluation of an evidence-based AD Risk Index exclusively based on self-reported information. The approach used for the development of the ANU-ADRI overcomes much of the sample bias that occurs when a measure is based on data from a single study both at the development and at the validation stage. When the c-statistic is estimated on the study from which an instrument is developed, it is often optimized to the characteristics of a specific study by trialling different cut-offs on predictor variables to obtain the best score. This applies generally to research in the development of risk assessment tools in medicine and health, and not just to dementia. Our findings demonstrate the value of evaluating different risk assessment tools on the same cohorts in order to evaluate their potential validity in different contexts. The c-statistics for this validation study compare favourably with those of widely used instruments in other fields of medicine even where they have been optimized to a single study. The Framingham risk index had a c-statistic of 0·79 [20] and one of the most commonly used breast cancer risk assessment indices reported a c-statistic of 0·58 [21]. It is also noteworthy that these and all dementia risk indices include age.

The ANU-ADRI includes 11 risk factors and four protective factors, yet the evaluation samples included a maximum of only eight to ten of these. This is in part due to the older age of the cohorts which means that BMI and cholesterol could not be included as predictors because the evidence for these relates only to midlife. Despite this potential limitation, adequate results of c-statistics ranging from 0.64 to 0.74 were obtained. Findings from the present study may underestimate the sensitivity of this tool in identifying individuals at increased risk of dementia when used in younger cohorts or cohorts where more risk and protective factors have been measured. The KP cohort included participants born before 1912 and was older than the other two validation samples, which is the likely explanation for the lower predictive validity of the ANU-ADRI within this cohort. Although individuals with baseline dementia were excluded from the KP study, it is possible that the sample included participants with Mild Cognitive Impairment (MCI) which again would reduce the sensitivity of the analyses.

When a common set of risk and protective factors were used to estimate the ANU-ADRI accuracy across cohorts there were variations in the c-statistics. The largest decrement was seen in MAP, and was explained by removal of the protective effect of cognitively stimulating activities which was unique to MAP. In contrast, there was very little change in the c-statistics for CVHS after removing fish intake, and depression suggesting effects of these factors are explained by other measures in the index.

Another dementia risk index for late-life was developed from the CVHS dataset and included clinical, performance and lifestyle data (n = 3608, mean baseline age 76 years) [10]. When evaluated on the same dataset it had a c-statistic of 0·82 and a short form of this risk assessment evaluated on the same dataset had a c-statistic of 0.77 [19]. Age, poor cognitive performance, low BMI, APOE genotype, white matter hyperintensities on brain MRI, ventricular enlargement, thickening of the carotid artery, history of bypass surgery, slow physical performance and abstaining from alcohol were included in this risk index. It is likely that several of the risk factors included in this index are not independent of AD, as neurological changes such as ventricular enlargement, and poor cognitive performance may be a consequence of the disease and be evident prior to clinical diagnosis. Hence, this index is not directly comparable with the ANU-ADRI or the CAIDE index. Integrating instrumental or laboratory assessments like MRI data, APOE genotype, history of coronary artery disease, and diagnostic assessments such as cognitive tests, would almost certainly increase the predictive power of a risk index for AD. This may be desirable in clinical settings. However, the purpose of the present study was to develop an index for use in the population who are asymptomatic to enable population level prevention strategies and interventions.

The dementia risk index developed for the CAIDE study includes age, sex, education, hypertension, high cholesterol and obesity and predicted dementia risk over a 20 year follow-up period [5] with a c-statistic of 0·77 on the study from which it was developed. The CAIDE was developed using data on risk factors in midlife and there is no comparable measure that has been developed on risk factors measured in older adults. We evaluated the CAIDE as a comparison measure for the ANU-ADRI because it included comparable risk factors. Moreover, it is valuable to evaluate whether a risk index developed on midlife adults is effective in predicting dementia when applied to older cohorts as this would provide information about the need for development of new indices such as the ANU-ADRI.

The analysis of CAIDE risk score showed that it did not have high c-statistics when used on older cohorts, and suggests that its use is likely to be optimal when applied to midlife cohorts. It is also possible that the weights developed for CAIDE are study specific. Our own analysis of the CVHS study comparing results for those aged under 70 by quartile of ANU-ADRI score, with those aged 70 or over, showed general similarities in the proportions of dementia cases in each age-group (Table 5). Nevertheless, due to the diversity of risk factors and their own developmental trajectories, there is a need for development of alternative instruments that are applicable at different ages, or for instruments that incorporate the flexibility to moderate risk scores based on age.

From an epidemiological perspective, each cohort study may be considered as an individual observation within the population of cohort studies, and the best estimates of weights for risk factors will be derived from the population. The most relevant weights are therefore derived from meta-analyses of findings from cohort studies reporting associations between risk factors and dementia. To date there remain risk factors where there are insufficient published studies to derive reliable estimates, and it is also possible that estimates vary according to age but that this information is not captured in publications which report a single risk estimate. Hence it is likely that considerable refinement of the ANU-ADRI is possible.

Limitations of the present study were that the evaluation samples were relatively old at their baseline assessment and would have been affected by selection bias in their initial recruitment. Hence the validity and ultimate utility of the ANU-ADRI needs to be further evaluated on younger cohorts. This will be done as datasets become available. The need to exclude those with prevalent dementia at baseline for this validation exercise also may result in a selected sample which would represent lower rates of dementia than those found in the population. We expect the c-statistics to be higher when risk factors are assessed in middle age [19]. The older ages of the validation samples precluded inclusion of risk factors such as high cholesterol and overweight or obese BMI, which are risk factors for dementia only when measured at middle age. The length of follow-up of the evaluation studies was relatively short, given that a number of risk factors included in the ANU-ADRI have been shown to be predictive from midlife in individual cohort studies [2]. Isolated findings from the KP, MAP and CVHS cohorts were also included in some of the meta-analyses that were used to derive effect sizes for the ANU-ADRI hence the validation samples were not purely independent of the measure development. For example, the KP contributed data to the pooled effect size for social networks. Most of the studies included in meta-analyses of risk factors for dementia control for covariates e.g. [22] yet there remains a possibility that the risk scores are influenced by residual confounding. This limitation would apply to other risk indices in the literature. The lack of biomarkers of AD and cerebrovascular disease in the cohorts examined means that it is likely that the extent of cerebrovascular disease and premorbid AD in the cohorts were underestimated. The close correspondence between the AUCs for dementia and AD suggests that the measure applies more broadly to dementia risk although the measure was based entirely on effect sizes derived from studies of AD.

The ANU-ADRI is based on the current knowledge of risk factors for AD. For example, in systematic reviews, hypertension has not been shown to be an independent risk factor for AD [23] yet it is possible that this is due to methodological factors influencing the measurement of hypertension in cohort studies. Hence limitations in the current knowledge base on risk factors for AD will also be limitations of the ANU-ADRI and any similar risk index.

Strengths of this study included the validation of the ANU-ADRI on cohorts from varied geographical locations with different population characteristics, supporting the generalizability of the instrument. The ANU-ADRI can be delivered via the internet, providing a virtually free method of risk assessment that may be used in universal population-health initiatives and by clinicians. The ANU-ADRI represents an advance in assessing risk profiles for dementia by including a wider range of factors than previous measures, and drawing on a wider range of literature in its development and evaluation. The methodology used to develop the ANU-ADRI enables it to be updated and improved as new information becomes available. For example, there is increasing evidence that exposure to air pollution increases the risk of cognitive decline [24] and possibly dementia. Future research evaluating the ANU-ADRI scores against biomarkers will enable refinement of the measure and clarification of the extent to which it measures AD risk specifically, compared to all cause dementia. Finally, the ANU-ADRI is available at http://anuadri.anu.edu.au for use by individuals and researchers.
